# Current Progress, Applications and Challenges of Multi-Omics Approaches in Sesame Genetic Improvement

**DOI:** 10.3390/ijms24043105

**Published:** 2023-02-04

**Authors:** Huan Li, Muhammad Tahir ul Qamar, Li Yang, Junchao Liang, Jun You, Linhai Wang

**Affiliations:** 1 Key Laboratory of Biology and Genetic Improvement of Oil Crops, Ministry of Agriculture, Oil Crops Research Institute, Chinese Academy of Agricultural Sciences, Wuhan 430062, China; 2 Integrative Omics and Molecular Modeling Laboratory, Department of Bioinformatics and Biotechnology, Government College University Faisalabad (GCUF), Faisalabad 38000, Pakistan; 3 Jiangxi Province Key Laboratory of Oil Crops Biology, Crop Research Institute, Nanchang Branch of National Center of Oil Crops Improvement, Jiangxi Academy of Agricultural Sciences, Nanchang 330000, China

**Keywords:** sesame, genomics, methylomics, transcriptomics, proteomics, metabonomics

## Abstract

Sesame is one of the important traditional oil crops in the world, and has high economic and nutritional value. Recently, due to the novel high throughput sequencing techniques and bioinformatical methods, the study of the genomics, methylomics, transcriptomics, proteomics and metabonomics of sesame has developed rapidly. Thus far, the genomes of five sesame accessions have been released, including white and black seed sesame. The genome studies reveal the function and structure of the sesame genome, and facilitate the exploitation of molecular markers, the construction of genetic maps and the study of pan-genomes. Methylomics focus on the study of the molecular level changes under different environmental conditions. Transcriptomics provide a powerful tool to study abiotic/biotic stress, organ development, and noncoding RNAs, and proteomics and metabonomics also provide some support in studying abiotic stress and important traits. In addition, the opportunities and challenges of multi-omics in sesame genetics breeding were also described. This review summarizes the current research status of sesame from the perspectives of multi-omics and hopes to provide help for further in-depth research on sesame.

## 1. Introduction

Sesame (*Sesamum indicum* L.), one of the oldest oil crops, is cultivated in tropical and subtropical regions of Asia, Africa and South America. At present, the total area of sesame in the world is about 13.96 million hectares, with an annual production of 6.8 million tons (2020, FAO). As one of the top four sesame producers, China has accounted for 9.7% of the world’s total sesame production in the past 10 years (2011–2020, FAO). Sesame seeds have the highest oil content, containing 45% to 63% oil [1], and are also rich in protein, vitamins, and unique antioxidant compounds, such as sesamin and sesamolin, which make it a healthy and nutritious food [2].

In the past, sesame was regarded as an orphan crop because it was hardly supported by science and industry; therefore, it lags behind other major oil crops in terms of genetic improvement. Moreover, sesame usually grows in harsh environments and is subject to various abiotic and biotic stresses, which seriously affect its productivity [3]. Due to the lack of tools and resources to aid a deep understanding of the potential molecular background of important agronomic traits, limited progress can only be made in these directions through traditional breeding, resulting in the slow pace of sesame improvement strategies. However, with the increase in knowledge about the diet and health benefits of sesame, the demand for sesame seeds and oil has increased sharply. Therefore, excellent genetic and breeding methods need to be solved urgently.

With the development and the application of multi-omics research methods, people are gradually understanding the genetic structure of the sesame genome and the relationship between the genotype and phenotype. In particular, the decoding of the sesame nuclear genome has led to the development of various genome resources, including the construction of genetic maps, pan-genome study, genome comparison and molecular marker development. At the transcriptome level, it is widely used to clarify the differential expression of biotic and abiotic stress response genes in different sesame genotypes, as well as the function of genes during the development of tissues and organs. Methylomics, proteomics and metabonomics are emerging omics technologies in the post-genomic era, which can resolve the role of sesame in different environmental conditions at different molecular levels. Here, we explored the candidate genes for key agronomic traits related to sesame development through multi-omics technology. These agronomic traits include high oil content, waterlogging and drought tolerance, disease resistance, heat and cold tolerance, and high yield, providing new strategies for sesame genetic improvement.

Due to the limited teams engaged worldwide in sesame research, concerted efforts are needed to integrate these resources into sesame breeding to improve quality and productivity. This review focuses on the latest progress in the field of “omics” in sesame, and traces the research progress from the perspective of genome, methylome, transcriptome, proteome and metabolome. It is hoped that, through these existing resources, our review will provide assistance in the wide and deep study of sesame.

## 2. Genomics

**Figure 1:**
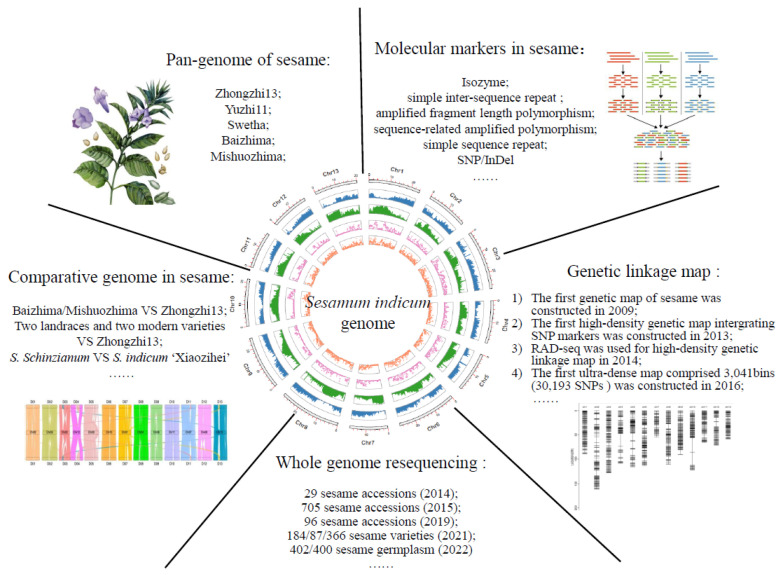
The applications of genomic approaches in sesame, including molecular markers, genetic linkage map, whole genome resequencing, comparative genome and pan-genome.

With the rapid development of second- and third-generation sequencing technologies, researchers have sequenced the genomes of cultivar sesame, the nuclear genome and the plastid genome, in order to facilitate the exploitation of molecular markers, the construction of a genetic map, and accelerate the construction of various genetic maps by whole genome resequencing and the study of a pan-genome in sesame (Figure 1). These could help us to obtain excellent genes that are related to oil synthesis and anti-oxidation, increase the breeding technology in sesame, improve the quality of sesame varieties, and understand the physiological mechanisms of some important traits in sesame.

### 2.1. Genome Sequencing in Sesame

#### 2.1.1. The Nuclear Genome in Sesame

Zhongzhi 13 is a variety of sesame that is suitable for the Yangtze–Huaihe valley, and its oil content is 59%. In 2014, the Oil Crops Research Institute of the Chinese Academy of Agricultural Sciences collaborated with BGI, University of Copenhagen and other units to complete the genome of Zhongzhi 13 into 16 linkage groups through second-generation Illumina sequencing [4]. A high-quality genome was assembled with a size of 274.9 Mb, containing approximately 27,148 genes, of which 84% of these genes were annotated. In addition, the total number of oil synthesis genes was small (only 708 genes), but the *LTP1* gene, related to oil transportation, was significantly expanded in soybean; meanwhile, the *LOX* and *LAH* genes, related to oil degradation, were notability contracted. In 2020, the genome of Zhongzhi 13 was reassembled by the third-generation Pacbio sequencing data and Hi-C data, and it had a 292.3 Mb full-length genome and almost 28,406 coding genes [5]. Compared with the previous one, the new Zhongzhi 13 assembled genome was highly complete and contiguous. Based on this high-quality reference genome, changes in the chromatin structure between normal growth and dark-treated seedlings were discovered in sesame. The differentially expressed genes (DEGs) induced by dark treatment were enriched in altered topologically associated regions and differential contact regions of the genome, suggesting that the chromatin organization was related to the gene transcription of dark treatment in sesame.

Since then, several genomes of other sesame accessions have been sequenced and assembled (Table 1). The genomes of the Indian cultivar “Swetha” (National Bureau of Plant Genetic Resources) and the modern cultivar “Yuzhi 11” (Henan) were completed in 2015 and 2016, respectively. Both were assembled at the scaffold level, with a genome size of 340 Mb and 211 Mb, respectively. In addition to the above three genomes, the literature also reported two sesame landraces, *S. indicum* cv. Baizhima and Mishuozhima [6], whose assembled genomes were 267 Mb and 254 Mb, respectively. There were two other sesames, Baizhima and Xiaozihei, assembled in this year, and the genomes were 309 and 305 Mb, respectively [7,8]. The above sequenced genomes could help one to deeply understand the genetic basis of sesame diversity, and also provide a large number of genes and variation profiles related to important traits and environmental adaptation, and promote the progress of sesame at a molecular level.

#### 2.1.2. The Nuclear Genome in Sesame

The chloroplast is a vital plastid existing in plants and algae that is involved in important functions, such as plant photosynthesis and the related biological processes; it is a typical tetrad structure, including two inverted repeats (IRs), a large single copy region (LSC) and a small single copy region (SSC) [9]. In plants, the circular chloroplast genome size is usually 120–160 kb, and is always used for species evolutionary analysis, genetic modification and breeding in crops [10]. The complete chloroplast genome of black-seed sesame (Ansanggae) was assembled in 2012; it was approximately 153,324 kb and contained 114 genes (80 coding genes (Table 2), 30 tRNA genes, and 4 rRNA genes) [11]. Furthermore, the whole chloroplast genome of white-seed sesame, Yuzhi11, was sequenced; it was 153,338 kb and 114 single-copy genes were identified, in detail: coding genes, tRNA genes and rRNA genes accounted for 50.44%, 1.84% and 5.90%, respectively. There were only 14 differences existing within the nucleotide sequences of homopolymers between these two chloroplast genomes [12]. In a word, these studies not only promote our understanding of the genome characteristics in sesame, provide important genetic information for sesame genetic improvement and improve breeding quality, but also accelerate the study of phylogeny and the evolution of sesame.

At present, the mitochondrial genome of sesame has not been submitted in public databases, and only the extraction technology of mitochondrial DNA has been mentioned for sesame. We look forward to the early completion of mitochondrial assembly with the progress of science and technology.

### 2.2. Applications of Sesame Genomics

#### 2.2.1. Molecular Marker in Sesame

Molecular markers are genetic markers based on the variation of nucleotide sequences in genetic material among individuals, and are a direct reflection of genetic polymorphism at the DNA level. The study of molecular markers not only accelerates the progress of modern plant breeding, but also improves the genetic gain and could shorten the breeding cycle in many crop species [13]. Before the development of the next-generation sequencing technique, isozymes were used to evaluate the genetic diversity of the sesame germplasm. Díaz and Layrisse [14] used isozyme to identify lower total genetic diversity among sesame populations (India, Korea, West Asia, Africa, China-Japan, and Central Asia), and found a unique allele in the China–Japan germplasm. With the development of sequencing technology, several universal molecular markers were applied for sesame genotyping and breeding, such as simple inter-sequence repeat (ISSR), amplified fragment length polymorphism (AFLP), sequence-related amplified polymorphism (SRAP), and microsatellites or simple sequence repeats (SSRs) in the genetic diversity analysis of sesame for its improvement [15]. For instance, a set of 34 ISSRs established the genetic diversity of 94 sesame germplasms with morphological and geographical diversity, and supported the allocation of germplasm lines to five major clusters and 21 subclusters based on allele data [16]; in total, 140 AFLPs were applied to analyze the molecular genetic diversity of 137 Turkish sesame genotypes (129 accessions and 8 cultivars), and found that 13 accessions (10%) had as many seeds as Kepsut99 or more, demonstrating that they contained useful alleles that could be used to improve seeds per capsule [17]. SRAP markers were utilized to assess genetic diversity in 52 sesame germplasms. Then, the combination of 17 of these primers yielded a large number of alleles (365), with 100% polymorphism [18]; about 38 SSR markers were supplied to assess genetic diversity in 25 sesame germplasms, which were collected from five regions in northern Ghana and Africa. The analysis of variance showed that there was a significant difference between the number of capsules in plants and the number of seeds per capsule. Additionally, the molecular markers had divided all these accessions into five different groups, and a total of 410 alleles were detected [19]. Considering that the polymorphism of the universal molecular markers in samples is unstable, the species-specific DNA markers (single nucleotide polymorphism (SNP) markers and insertion/deletion (InDel) markers) have been used as substitutes in the genetic research of sesame in recent years. Wei et al. [20] utilized three RNA-seq datasets to construct a reference transcriptome using de novo assembly, and found a total of 7450 SNPs and 362 InDels among the 37,646 transcripts, with complete open reading frames. As whole genome resequencing develops, the application of SNP/InDel in sesame is increases [21,22]. The molecular marker technology of sesame has made rapid progress in the past decade, but it has not yet integrated modern biotechnology tools into traditional breeding to improve crops.

#### 2.2.2. High-Density Genetic Linkage Map of Sesame

A genetic linkage map could reveal the relative position and linear arrangement of genetic markers on chromosomes, which is an important means of gene location and germplasm innovation [23]. It is generally known that the first genetic map of sesame was constructed by using an F2 population COI1134 × RXBS in 2009 (Table 3). A total of 220 markers (8 EST-SSRs, 25 AFLPs, and 187 RSAMPLs) were located in 30 linkage groups (LGs), which covered approximately 936.72 cM; the average marker spacing was 4.93 cM [24]. Subsequently, this map has been improved through 653 markers (30 EST-SSRs, 50 AFLPs, and 573 RSAMPLs), located on 14 LGs with a length of 1216.00 cM, and QTLs for seed coat color have been identified for the first time in sesame [25]. Zhang et al. [26] constructed a genetic map with 70 marker loci grouped into 15 LGs, and identified 6 QTLs related to waterlogging tolerance at the flowering stage. Wang et al. [27] used 424 SSR markers to construct a genetic map that had 13 LGs and approximately 1869.8 cM, and 14 QTLs with charcoal rot disease resistance were detected. Using the same genetic map, Xu et al. [28] identified QTLs for sesamin and sesamolin content variation. With the development of sequencing technology, the simplified genome sequencing techniques were used for the construction of high-density genetic linkage maps in sesame. The first high-density genetic linkage map was constructed by using an F2 population Shandong Jiaxiang Sesame × Zhongzhi 13 through the specific length amplified fragment sequencing (SLAF-seq) in 2013 [29]. An average marker spacing of 1.20 cM was found on the map, which contained 1233 SLAF markers located on 15 LGs. Subsequently, the restriction-site associated DNA sequencing (RAD-seq) method was utilized to construct a high-density genetic map with 1230 markers, and several grain yield-related QTLs were identified [21]. Up to now, eight more genetic linkage maps have been constructed in sesame with SLAF-seq, RAD-seq, ddRAD-seq or GBS [30,31,32,33]. These genetic maps have been used to identify QTLs related to yield and yield components, seed quality, disease resistance, stress tolerance, and other traits in sesame. For example, Wang et al. [34] applied RAD-seq to construct a high-density sesame genetic map with 1522 bins on 13 LGs, which equaled the number of sesame chromosomes for the first time. Using this bin map, several QTLs were identified for plant height and seed coat color. Uncu et al. [35] utilized the genotyping by sequencing (GBS) approach to construct a genetic map with 432 markers (420 SNPs, 12 SSRs), which mapped to 13 LGs with a length of 914 cM. Mei et al. [36] used SLAF-seq to develop a high-density genetic linkage map in sesame, which had 9378 SLAF markers and located 13 LGs. Based on this new genetic map, the basal branching habit *(SiBH*) and axillary flowers per leaf (*SiFA*) genes were mapped to LG5 and LG11, respectively. Using the whole genome resequencing strategy, Zhang et al. [37] constructed the first ultra-dense SNP map in sesame, which comprised 3041 bins (containing 30,193 SNPs) on the 13 LGs, with an average marker density of 0.10 cM per SNP or 0.98 cM per bin marker. After that, four more ultra-dense genetic linkage maps were constructed and used for the high resolution mapping of QTLs in sesame [38,39,40,41]. A high-density genetic linkage map, consisting of 19,309 markers (3030 SNPs and 16,279 InDels), was constructed for analyzing the QTLs associated with yield components and seed minerals. S-91 was found to be a favorable allele (higher nutrient concentration) at all four loci (LG6, LG8, LG11, and LG16) [39]. Liang et al. [40] constructed an ultra-density linkage map with 1354 bin markers (538,090 SNP/InDel variants), and 8 stable QTLs, related to drought stress tolerance, were detected. Therefore, a good genetic map can provide a framework for genetic analysis and the gene location of multiple traits, as well as quantitative trait locus analysis, map-based gene cloning, and genome sequence assembly.

#### 2.2.3. Whole Genome Resequencing in Sesame

Whole-genome resequencing (WGS) is a method used to sequence the genomes of different individuals in species, based on a known genome reference, and to analyze the differences between individuals and populations on this reference [42]. Since the first sesame genome was completed in 2014, studies on resequencing have been increasingly carried out in sesame (Table 4). For instance, 29 sesame accessions from 12 countries, with an oil content between 48.6% and 59.8%, were chosen for WGS, and a total of 2,348,008 SNPs were identified. The oil-related genes with a high population diversity were significantly enriched in two biological engineering scenarios of lipid transport and localization [43]. A total of 705 sesame accessions were resequenced to identify sequence variations, and 549 associated loci were found for 56 agronomic traits [44]. Based on these data, several analyses have been carried out: ① In total, 490 accessions were selected for revealing the genetic variants leading to salinity and drought tolerances at germination stage. A total of 13 and 27 potential candidate genes were observed for drought and salt tolerance indexes, respectively [45]. ② A genome-wide association study (GWAS) was performed for traits related to drought tolerance through 400 accessions, and it was found that *SiSAM* conferred drought tolerance by modulating polyamine levels and ROS homeostasis [46]. ③ Through 705 accessions, 547 loci were significantly associated with the 39 seed yield-related traits, then 48 genes were found to control capsule length and capsule number traits [47]. ④ In total, 327 accessions were performed in the GWAS on seven root traits, and 32 candidate genes were identified [48]. ⑤ Based on 450 accessions, three significant associations, located on LG2, LG4 and LG6, were identified for melatonin content, and the overexpression of SiWRKY67 was found to potentially promote the melatonin content in the hairy roots [49]. ⑥ A total of 433 SNP loci, associated with variations in the primary metabolite contents, were identified by 412 sesame accessions, and 10 candidate causative genes were observed for variation in monoacylglycerols, fatty acid, asparagine, and sucrose contents [50]. A total of 96 accessions were utilized to detect loci associated with tocotrienol contents, and found that one locus on LG8 was a significantly associated with γ-tocopherol in sesame seed [51]. The morpho-agronomic traits under the Mediterranean climate conditions were analyzed by 184 genotypes, and 50 signals associated with these traits were identified [52]. Linkage mapping and GWAS were performed on 87 sesame accessions for phytophthora blight resistance, and 29 significantly associated SNPs on LG10 were identified. The gene SIN_1019016 was found to show a significantly higher expression in the resistant parent [31]. The seed coat color in 12 environments was evaluated by 366 sesame accessions, and 224 SNPs were identified for three seed coat color space values. In addition, 92 candidate genes were observed for the vicinity of the 4 reliable and stable peak SNPs [53]. The mature seed samples of 402 sesame accessions were selected in two different environments. A total of 33 significant SNP loci, associated with the four phytosterols, were detected, and 37 candidate genes were selected for future studies [54]. Gas chromatography was utilized to analyze natural changes in fatty acid composition and oil content in 400 sesame accessions, and 43 loci, related to fatty acid and oil content, were identified. Then, the *SiTPS1* gene was found to be a key regulatory gene for fatty acid biosynthesis and triacylglycerol metabolism in sesame [55]. Dossou et al. identified 5.38 and 1.16 million SNPs and InDels from 410 sesame accessions, and found 17 and 72 SNP loci for the sesamin and sesamolin variation, respectively [56]. Therefore, the application of WGS in plants, along with the quick development of sequencing technology, can provide high-quality SNPs for phylogenetic and population analysis, and those results could contribute to subsequent molecular breeding, functional and evolutionary research.

#### 2.2.4. The Application of Comparative Genomics in Sesame

Comparative genomics refers to the study of gene function, expression mechanism and species evolution by comparing known genes and genome structures based on the genome genetic map and sequencing. Previous studies have reported that Sesamum can be divided into three types according to the number of chromosomes, that is, 2 *n* = 26 (such as *S. indicum*, *S. alatum*), 2 *n* = 32 (such as *S. protratum*, *S. angolense*) and 2 *n* = 64 (such as *S. radiatum*, *S. schinzianum*) [57]. The basic chromosome number of Sesamum is X = 8 and 13, where X = 13 may be caused by ancient polyploidy. At present, researchers primarily focus on the study of sesame genome comparison in two aspects: interspecific comparison and intraspecific comparison. For the study of interspecific comparison, they focus on the differences between the genomes of different sesame varieties. To identify sesame landraces and genomic variation among cultivars, the genomes of landrace Baizhima and Mishuozhima were selected to compare with Zhongzhi 13 reference genome, and identified a total of 1,332,025 SNPs and 506,245 InDels, respectively. Then, these genomic variants were applied for QTL mapping, and the melanin synthesis gene *PPO* was found to be a candidate gene for sesame seed coat color [58]. Additionally, two landraces and three modern varieties of sesame were compared, and it was observed that modern varieties from China and India had significant genomic variations. The specific gene families of modern sesame varieties (2080 gene families and 13,094 variety-specific genes) mainly contained genes related to yield and quality, while that of landraces (552 gene families and 2796 variety-specific genes) included genes related to environmental adaptation [6]. Recently, third generation sequencing of PacBio Sequel II platform and Hi-C data were utilized to assemble two high-quality sesame genomes (wild tetraploid sesame and cultivated diploid elite sesame). Some differences in the number of chromosomes were identified between these two genomes, but the high collinearity of genes illustrated the conservation of gene evolution and the stability of the genome structure [8]. As for the intraspecific comparisons, it is the main method of comparative analysis among the same population of the sesame. Many variations and polymorphisms could be observed. In a nutshell, the genomic studies of different sesame varieties may provide extensive genomic resources for the biology, genome diversity and evolution of sesame.

#### 2.2.5. The Pan-Genome of Sesame

**Figure 2:**
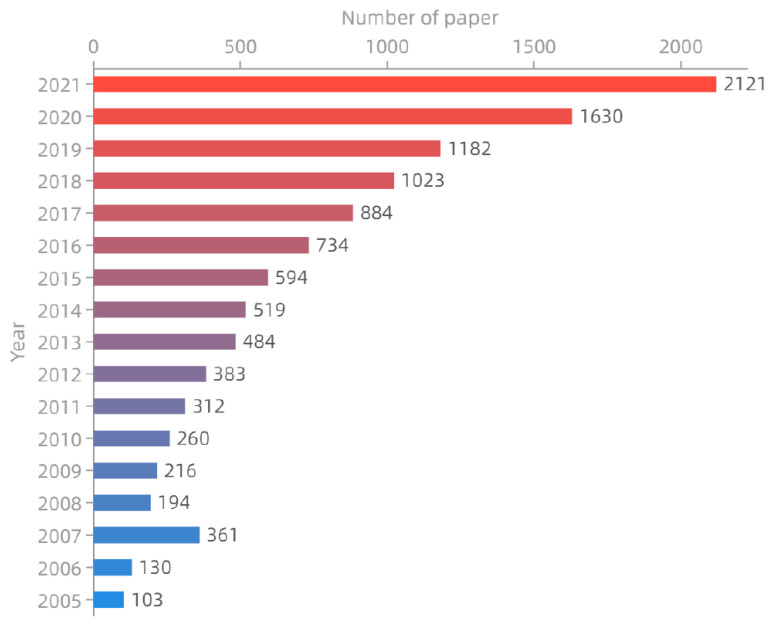
The summary of plant pan-genome papers in seventeen years. We have collected the data of the articles published in the plant pan-genome from 2005 to 2021 (Europe PMC). Especially in the last ten years, it has developed rapidly.

The pan-genome is the collection of all the genomic information within a species, covering more genetic diversity than a single reference genome. This concept was first proposed in the study of *Streptococcus agalactiae*, including the core genome that existed in all strains and the dispensable genome that existed in some strains or unique strains [59]. The earliest study of a pan-genome in plants was in 2007, when, comparing the genomic regions of two inbred lines of maize, it was found that the core genome and the dispensable genome were significantly different in the characteristics of DNA sequence variation in the genome of the species [60]. Since 2014, the construction of pan-genomes in maize and rice has started the era of plant pan-genome research [61,62]. Figure 2 shows the statistics data of the articles published in the plant pan-genome from 2005 to 2021 (Europe PMC). The application of the pan-genome includes combining GWAS data to effectively improve its accuracy and capture more complete genetic variation information; structural variation may affect the transcriptional regulation and the expression of a gene by changing the sequence of gene regions or gene flanking regions; the pan-genome can provide more complete and extensive support for the study of plant evolution and domestication because of the complete genome diversity of the population genomes [63].

At present, the pan-genome has been carried out in the research of sesame. In 2019, the first pan-genome of five sesame varieties, including Zhongzhi 13, was constructed [6]. The entire pan-genome was about 554.05Mb, containing 258.79Mb of core genome and 295.26Mb of dispensable genome. After homologous alignment, 26,472 orthologous gene clusters were found, of which 15,409 (58.21%) were core gene clusters, while the remaining were 11,063 gene clusters (41.79%) and 15,890 breed-specific genes. The high proportion of dispensable orthologous gene clusters and cultivar-specific genes highlights the diversity of the sesame genome, which, in turn, may contribute to its phenotypic diversity and local adaptation. Overall, the study of the pan-genome provides a useful resource for identifying genomic variation in different sesame lines, and understanding genetic diversity and phenotypic variation.

## 3. Methylomics

**Figure 3:**
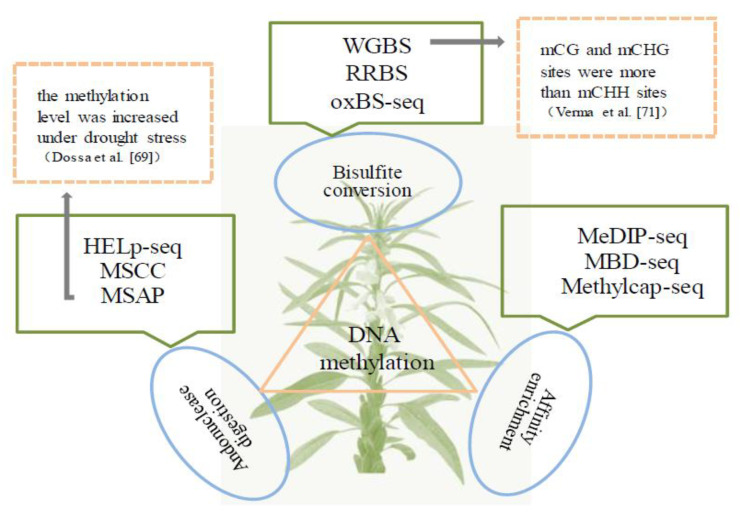
The main methods of DNA methylation in sesame. It mainly contains whole genome bisulfite methylation sequencing (WGBS), oxidative bisulfite sequencing (OxBS-seq), reduced representation bisulfite sequencing (RRBS), methylation DNA immunoprecipitation sequencing (MeDIP-seq), HpaIItiny fragment enrichment by ligation-mediated PCR(HELP-seq), and so on.

DNA methylation is a heritable epigenetic mechanism. There are five general sequencing methods of DNA methylation: whole genome bisulfite methylation sequencing (WGBS), oxidative bisulfite sequencing (OxBS-seq) [64], reduced representation bisulfite sequencing (RRBS) [65], methylation DNA immunoprecipitation sequencing (MeDIP-seq) [66], and HpaIItiny fragment enrichment by ligation-mediated PCR(HELP-seq) [67], which are applicable to solute different research directions (Figure 3). DNA methylation can effectively regulate genome stability, and the release of the sesame genome laid the foundation for DNA methylation analysis. Furthermore, previous studies have shown that epigenetic modifications are widely involved in the abiotic stress responses of plant and are incredibly important for their adaptation to changing environments [68]. As one of the most substantial epigenetic modifications, the role of DNA methylation in regulating and responding to abiotic stress has attracted extensive attention. 

At present, whole genome bisulfite sequencing technology is applied to carry out methylation-related studies on sesame. Dossa et al. [69] utilized 25 primer combinations to evaluate cytosine methylation patterns in the root of the drought-tolerant and waterlogged sesame genotype under three different conditions (control, stress, and recovery). The results demonstrated that the methylation level was significantly increased under drought stress, while the methylation level decreased under waterlogging stress in the stress treatment. During the recovery phase, methylation levels tended to reach those observed under control conditions. Singh et al. [70] chose the regulatory genes (*UFO*, *FLO*, *FLT* and *SUP*), which were involved in flower development and their orthologous gene regions, for primer design in sesame. Then, the differences in their cytosine methylation status between healthy and infected conditions were detected by McrBC assay, but there was no differential methylation. Verma et al. [71] reported upon the DNA methylation changes in healthy and phytoplasma-infected sesame by using the whole genome bisulfite sequencing approach. In four sesame samples, most of the mCG and mCHG sites at methylation levels were above 90% (unimodal distribution), while the mCHH sites were more widely distributed than the mCG and mCHG sites. Additionally, the largest position in the CHH context had 40–60% methylation, while about 20% of the positions had methylation levels between 80–100%. In particular, most of the mCHH methylation levels were between 10% and 40%. It is commonly known that the changes in DNA methylation could enrich the diversity of species and enhance the environmental adaptability of plants. Thus, the study of the dynamics of DNA methylation under abiotic stress can contribute to improving the resistance and stress ability of sesame under different environmental conditions by using epigenetic variations.

## 4. Transcriptomics

**Figure 4:**
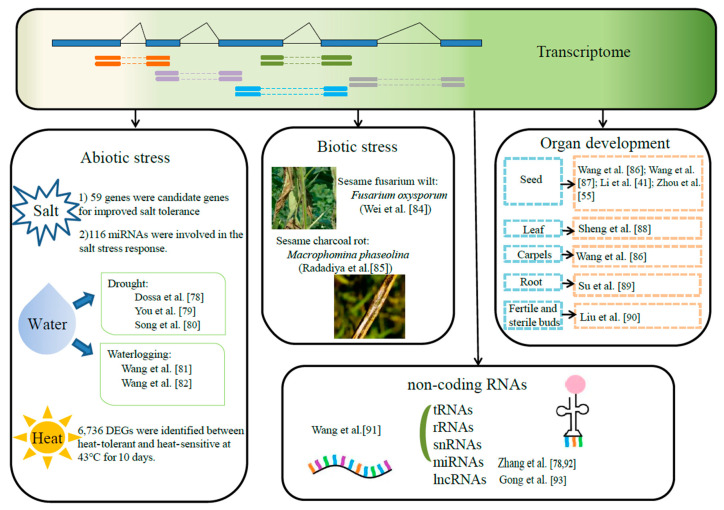
The applications of transcriptomics approaches in sesame. The research of sesame under abiotic stress is introduced from the aspects of salt, water and heat temperature [76,77,78,79,80,81,82,83]. The biotic stress illustrates two main sesame diseases: sesame fusarium wilt and charcoal rot [84,85]. In terms of organ development, seeds, leaves, carpels, roots, fertile and sterile buds are introduced [41,55,86,87,88,89,90]. It also introduces several non-coding RNAs, such as tRNAs, rRNAs, miRNAs, lncRNAs [77,91,92,93].

Transcriptomics is the study of gene expression and its regulation at the RNA level. Transcriptome is an inevitable link between genomic genetic information and biological function, and is one of the most active disciplines in the post genomic era [72]. Currently, the transcriptome sequencing technology has been widely utilized in basic research fields, such as medicine, drug development and agricultural science. With the completion of the whole genome sequencing of maize, Arabidopsis, rice, cotton, potato and other plants [73,74], researchers have focused on the application of RNA sequencing technology to study gene expression patterns under different environmental conditions or at different growth and development stages, in order to understand the regulation mechanism of gene expression and explore candidates related to specific traits [75]. In fact, sesame is an oil crop with a large planting area in China. As an important food and medicine, it plays a vital role in human life. Therefore, studying the gene expression pattern of sesame under specific conditions is very important for the development and utilization of sesame germplasm resources (Figure 4).

### 4.1. Application of Transcriptomics in Abiotic Stress

Abiotic stress is a general term for all abiotic conditions that are unfavorable to plant growth, such as salinity, water, extreme temperatures, heavy metals, ultraviolet radiation, etc. [94]. When plants face adverse growth conditions, abiotic stress will hinder their growth and productivity, and plants adapt to an unfavorable living environment by changing their own morphology, physiology and biochemical level. Recently, researchers have mainly focused on the study on the abiotic stress of sesame in salt, water and heat (Table 5).

#### 4.1.1. Salt Stress

Salt stress not only affects the external morphology, but also influences the internal physical and chemical properties of plants. It is commonly known that sesame is moderately tolerant to salt stress, and salt-tolerant varieties are urgently needed in the saline areas of the main sesame production countries. Through the comparison of two sesame genotypes, it was found that the germination and survival rates of salt-tolerant sesame were higher than those of salt-sensitive sesame. A total of 1275 and 1946 DEGs were identified in four treatment groups of salt-tolerant and salt-sensitive sesame, respectively. In total, 59 genes were highly up-regulated in salt-tolerant sesame after salt treatment, which were identified as candidate genes for improved salt tolerance in salt-sensitive sesame [76]. Subsequently, 18 small RNA libraries of salt-tolerant and salt-sensitive genotypes under control and salt-stress conditions were constructed, and 442 miRNAs were identified. Among them, 116 miRNAs were involved in the salt-stress response, and they showed different expression levels under salt-stress by analyzing the regulatory network of miRNA-mRNA [77]. These theoretical studies are crucial for cultivating new salt-tolerant sesame varieties and understanding the molecular mechanism of the adaptive response to salt stress.

#### 4.1.2. Water Stress

Water is an indispensable substance in life, and it has the greatest impact on plants. Through the response to drought between two sesame genotypes (tolerant and sensitive), Dossa et al. [78] observed that the tolerant genotype relied on well-functioning taproots that could provide water to the above ground tissues. In addition, the tolerant genotype strongly activated genes related to antioxidant activity, osmotic protection, and hormone signaling pathways. The candidate genes with a higher tolerance constituted useful resources for improving drought tolerance in sesame. You et al. [79] discovered a core set of drought-responsive genes by analyzing the transcriptome data of leaves from two sesame genotypes (drought-tolerant and drought-sensitive). They included 684 up-regulated and 1346 down-regulated genes, which were expressed differently in these two genotypes. Furthermore, Song et al. [80] performed polyethylene glycol (PEG)-induced osmotic stress to explore the transcriptional changes in the roots of drought-tolerant and drought-sensitive sesame. A total of 1251 and 541 genes were detected differentially expressed in PEG-treated and untreated roots, respectively. During osmotic stress, multiple members of the WRKY, bZIP, MYB and NAC families were also found to be over-expressed in roots of the drought-tolerant genotype.

Like most typical dry-land crops, sesame is highly sensitive to waterlogging stress, and soil waterlogging will lead to hypoxia and high CO_2_ in the roots, slow down photosynthesis, and ultimately damage the normal growth and yield of crops [95]. The comparative analysis was utilized to explore the waterlogging stress response in two sesame genotypes, and a total of 19,316 genes were expressed during waterlogging. Among these, 66 genes were found to be potential candidates for improving sesame tolerance to waterlogging [81]. Dossa et al. [96] treated the flowering sesame for 36h with water, and then drained them for 12 h. The samples were collected at 22 time points under the waterlogging/draining treatment and 10 time points under the control condition. Subsequently, 47 core waterlogging-responsive genes were identified through these transcriptome data. Moreover, a temporal transcriptional network model was constructed to predict the putative causal relationships between TFs and downstream waterlogging-responsive genes, and some interactions were verified by yeast one-hybrid assays [82].

Therefore, our research on drought and waterlogging stress is mainly in order to obtain changes in their plant morphology, physiology and biochemistry; this is to find out the mechanism of drought and waterlogging stress, and lay a foundation for subsequent research on sesame genetics and breeding.

#### 4.1.3. Heat Stress

Heat stress causes serious damage to almost all stages of growth in the plant. It not only hinders the growth and development of the plant, but also causes irreversible damage to intracellular homeostasis, and even leads to death [97]. Su et al. [83] identified a total of 6736 DEGs by treating the seedlings of two sesame cultivars (heat-tolerant and heat-sensitive). They found that the heat-tolerant genotype had 5526 DEGs, of which 2878 were up-regulated and 2648 were down-regulated, while the heat-sensitive genotype had 5186 DEGs, of which 2695 were up-regulated and 2491 were down-regulated. These DEGs included heat shock proteins related to stress tolerance, and genes related to carbohydrate and energy metabolism, signal transduction, endoplasmic reticulum protein processing, amino acid metabolism, and secondary metabolism. The high temperature climate of this summer seriously affected the growth of surface vegetation, leading to serious ecological problems in many places. Thus, it is more urgent to study related heat-tolerant genes and transcription factors at the transcriptome level, so that the sesame could better adapt to a high temperature climate. It provides a theoretical basis for the breeding of temperature-resistant sesame to adapt to the future global warming.

### 4.2. Application of Transcriptomics in Biotic Stress

Apart from abiotic stress, plant growth and development are also greatly affected by biotic stress. When plants are attacked by bacteria, animals and other stresses, they will change the expression of genes and enzyme activities in the body to complete the induction and transmission of these signals, and the realization of biological effects. Previous studies have been reported that there are more than 170 pathogens causing diseases in sesame, of which the most serious diseases are sesame fusarium wilt and charcoal rot [98]. The following studies are about these two diseases: two sesame varieties, resistant and susceptible genotypes, were infected by *Fusarium oxysporum*; the KEGG analysis indicated that the “phenylpropanoid biosynthesis” pathway may play a more vital role in infected sesame, while the differences in fusarium wilt symptoms between resistant and susceptible genotypes may depend on whether plants can activate this pathway in an efficient manner [84]; by sequencing the transcriptome of two sesame varieties with different incidence rates 72 h after infection with *Macrophomina phaseolina*, 1153 and 1226 DEGs were identified in the resistant and susceptible genotypes, respectively. The resistant genotype showed the highest expression of genes at 24 h post-inoculation (hpi), while the susceptible genotype illustrated those at 48 hpi. It is speculated that the biosynthesis of flavonoids, carotenoids and caffeine via the phenylpropanoid pathway and salicylic acid biosynthesis at 24 hpi may contribute to sesame charcoal rot resistance [85]. These studies on diseases will help us to deeply understand the disease resistance of sesame varieties, and provide a theoretical basis for scientifically preventing and controlling important diseases and ensuring a bumper harvest of sesame.

### 4.3. Application of Transcriptomics in Organ Development

Gene expression is tissue specific, and the study of gene expression in different tissues and organs at different developmental stages is of great significance for understanding plant development and regulatory mechanisms, and for the better transformation and utilization of plants [99]. Here, the basic information of gene expression in different tissues during the development stage is presented (Table 6). Wang et al. [86] sequenced the seeds and carpels of three high-oil and low-oil sesame cultivars, and found that the number of expressed genes tends to decrease in seeds, but that it fluctuates in the carpels 10 to 30 days post-anthesis (DPA). The enrichment of genes involved in lipid biosynthesis can distinguish between high-oil and low-oil genotypes (30 DPA), suggesting that oil biosynthesis in seeds plays a key role at later stages. Furthermore, Wang et al. [87] detected two sesame cultivars with different seed coat colors (Zhongzhifeng 1, white seeds; Zhongzhi 33, black seeds) at the developmental stages (5, 8, 11, 14, 17, 20, 23, 26 and 30 DPA). Using 5 DPA as the control, 11 to 20 DPA groups were used to identify candidate genes related to melanin biosynthesis in sesame. In total, 1572 up- and down-regulated DEGs were identified. In addition, Li et al. [41] constructed a QTL mapping of seed coat color in sesame, and 17 QTLs were detected on four linkage groups. In the meantime, the DEGs between a white- and a black-seeded variety were enriched significantly in 37 pathways. Zhou et al. [55] compared the oil content and fatty acid compositions between Zhongzhi 16 and Mishuozhima, and identified a total of 8404 DEGs in the seeds. Combined with WGAS, 20 candidate genes were identified and SiTPS1 was found as a key regulatory gene of fatty acids and triacylglycerol metabolism in sesame. 

Except for the most important seeds, other tissues have also been studied. Sheng et al. [88] combined QTL-seq and SSR marker mapping methods to identify 56 candidate genomic regions controlling leaf size in RIL populations, crossed between large-leaf and small-leaf sesame varieties. In total, 12 were identified as expressing lower and 14 as expressing higher in the large leaf parent at both the 8th and 20th leaf stages, respectively, and three candidate genes (SIN_1004875, SIN_1004882 and SIN_1004883) that are associated with leaf growth and development were revealed. Su et al. [89] studied the root of 40 different sesame varieties grown in soil and hydroponic systems, and found that similar genotypes usually clustered in small or large root groups. A total of 2897 DEGs were identified, and these genes were mostly enriched in flavonoid, phenylpropanoid and gingerol biosynthesis, and starch and sucrose metabolism, etc., demonstrating that these pathways play vital roles in the growth and development of sesame roots. Liu et al. [90] analyzed the sterile flower buds of two near-isogenic DGMS lines, and identified 1502 significant DEGs, of which 751 were up-regulated genes. Many DEGs were also found to be involved in ethylene, and jasmonic acid synthesis and signaling pathways; their expression was up-regulated or down-regulated in sterile shoots, respectively. However, most NAC and WRKY transcription factors related to DEGs were up-regulated in sterile shoots. These studies have laid a foundation for an in-depth understanding of genetic characteristics and the variability of different tissues in sesame, and will be utilized as genetic resources for the structural improvement of plants. 

### 4.4. Research on Non-Coding RNAs

The applications of transcription illustrated above were mainly related to coding proteins, and the following was the introduction of non-coding regions. Noncoding RNAs (ncRNAs) are RNAs that cannot be translated into proteins but have catalytic activity, such as tRNAs, rRNAs, snRNAs, miRNAs and lncRNAs [91];ncRNAs have important biological functions, ranging from regulating gene expression and principal cellular functions, to affecting genome structure. When the sesame genome was completely assembled, a total of 207 miRNAs, 870 tRNAs, 268 snRNAs, and 386 rRNA fragments were identified at the same time [4]. miRNA plays a vital role in the response to abiotic stress by the post-transcriptional regulation of target gene expression in plants. Zhang et al. [77] identified 351 known miRNAs and 91 novel miRNAs from 18 sesame libraries, and 116 miRNAs were found to be involved in the salt-stress response through the comparison between the salt-treated group and the control group. Subsequently, Zhang et al. [92] sequenced a total of 220 miRNAs in seed development and constructed a regulatory co-expression network through transcriptomics, small RNAs and degradome. The FAD2, LOC10515945, LOC105161564, and LOC105162196 genes were observed for regulating the accumulation of unsaturated fatty acid biosynthesis. LncRNA plays a major role in various biological regulation processes involving complex mechanisms, and in the regulation of the stress response. Gong et al. [93] identified a total of 2482 lncRNAs from different sesame genotypes under salt stress by high-throughput RNA sequencing, of which 599 were intergenic lncRNAs, 293 were antisense lncRNAs, and 786 lncRNAs had the potential to encode proteins. Most lncRNAs were expressed at low levels, and about 700 differentially expressed lncRNAs were characterized as salt responsive. Functional annotation showed that differentially expressed lncRNAs under salt stress may regulate protein coding genes involved in multiple important pathways, such as glycolysis/gluconeogenesis, flavonoid biosynthesis, biotin metabolism, and monoterpenoid biosynthesis. At present, the study of biological functions and the mechanisms of ncRNAs in plants is mainly focused on model plants, such as Arabidopsis [100,101]. The research on miRNAs and lncRNAs in sesame is still at the initial stage. It is expected that more new ncRNAs, mature technologies and the use of the mechanism of ncRNAs to promote molecular breeding will be found in the near future.

## 5. Proteomics

Proteomics is the study of the composition of and changes in proteins expressed in a cell or a tissue. By analyzing the composition, expression level and modification status of proteins, we could understand the interactions and relationships between proteins, study the disciplinarian of proteins regulating life activities at the overall level, explore the regulation of life activities and explain unknown life phenomena at the functional level. It has made outstanding contributions to exploring the expression mode of known genes and plant resistance to stress [102]. In recent years, the study of proteomics in sesame has only started, laying a foundation for further research. Jung et al. [103] used LTQ-FTICR MS/MS technology to draw a proteome map of sesame leaves grown under blank and waterlogged conditions. The waterlogging treatment at different growth stages influenced the morphological development of sesame, and the longer the waterlogging duration was, the slower the growth was. Under waterlogging stress, it considered the decrease in chlorophyll content to be the acceleration of chlorophyll degradation or the inhibition of chlorophyll synthesis. Zhang et al. [104] analyzed the phenotypic, physiological and proteomic changes in salt-tolerant and salt-sensitive seedlings induced by salt treatment. The salt-tolerant seedlings were greener and healthier than salt-sensitive seedlings under salt stress. A total of 872 salt-responsive proteins were identified in both two genotypes, most of which were enriched in biological processes related to multiple metabolic processes, including single organism metabolism (27.9%), carbohydrate metabolism (9.3%), carboxylic acid metabolism (5.7%) and cell lipid metabolism (4.8%). Using the label-free quantitative shotgun proteomics, Pamei and Makandar [105] identified 3457 and 1704 proteins from the asymptomatic (control) and symptomatic (phytoplasma infected) sesame samples, respectively. Among them, 571 differential abundant proteins (DAP) were found to potentially be involved in the interaction between asymptomatic and symptomatic. A total of 212 DAPs were up-regulated and 21 DAPs were significantly down-regulated in symptomatic samples, and the up-regulated DAPs were mainly related to protein phosphorylation, defense response, protein ubiquitination, ATP binding, protein binding and DNA binding activity, while the down-regulated DAPs were related to the transmembrane receptor, the protein tyrosine kinase signal pathway, wound response, plasmodesmata, peptide receptor activity, etc. Through different proteomics research methods, we could understand the protein composition, specific expression and functional differences behind the changes in traits under various stresses; this is an important theoretical basis for obtaining good stress-adaptive traits in sesame.

## 6. Metabolomics

Metabonomics is a new science that searches for the relationship between metabolites and biological changes in physiology and pathology through the quantitative analysis of all metabolites in organisms. The content of and change in metabolites could directly reflect the variations of plants, so it is more convenient than other omics technologies. Compared with genomics, transcriptomics and proteomics, metabolomics can further expand the differences at the metabolic level and metabolic pathways by starting with biological phenotypic variations. Metabonomics plays an important role in revealing the response of organisms to biotic or abiotic environmental irritation or interference, and the corresponding functional gene tagging and mining; it has become one of the key technologies in the study of the stress adaptation mechanisms of organisms at this stage [106,107].

### 6.1. Research on Abiotic Stress

The study of plant metabolomics is one of the core aspects of content in the whole research of metabolomics. It is generally known that plant metabolites have the characteristics of various types, complex structures and unstable contents, which provide ideal materials for studying the differences and synthetic pathways of metabolites in response to biotic and abiotic stress [108]. Zhang et al. [77] studied the salt-tolerant and salt-sensitive sesame genotypes, and found that the salt responsive genes in sesame were mainly related to the biosynthesis of secondary metabolites, carbohydrate metabolism, amino acid metabolism, plant hormone signal transduction and redox process related. The metabolome analysis under salt stress revealed that the accumulation of metabolites involved in stress was high in salt-tolerant sesame, further emphasizing the enhanced metabolism of amino acid metabolism, and sucrose metabolism in the salt-tolerant genotype. You et al. [79] have found that the most important metabolites accumulated by two sesame genotypes (drought-tolerant and drought-sensitive) under drought stress contain abscisic acid, amino acids and organic acids. Under stress conditions, the drought-tolerant genotype had higher levels of abscisic acid, proline, arginine, lysine, gamma-aminobutyric acid and allantoin, etc. In particular, the combination of transcriptomics and metabolomics revealed the important roles of amino acid metabolism, abscisic acid metabolism and signaling pathway in the drought tolerance of sesame.

### 6.2. Research on Important Traits

Plant growth and development is an extremely complex process, including the formation of various tissues and organs, color formation and quality formation. Because of this, plants require numerous genes and metabolites to perform their functions, so elucidating a specific regulatory mechanism is a monumental challenge, and metabolome analysis methods have been widely used in exploring the process of plant growth and development. Wang et al. [109] applied a novel metabolomic strategy to detect different metabolite levels in black and white sesame seeds, and obtained 557 metabolites for the structure and content, of which 69 metabolites were discovered to be significantly different between two sesame genotypes. From 20 selected metabolites, and it was observed that there were some differences in metabolic pathways, such as phenylalanine biosynthesis, tyrosine metabolism and riboflavin metabolism. In addition, Dossou et al. [110] also utilized an LC-MS/MS-based widely targeted metabolomics analysis to study the diversity and variability of metabolites in sesame seeds of different colors (black, brown, yellow, and white). A total of 671 metabolites were identified and it was found that the antioxidant activities of the seeds increased with the seed coat darkness. In fact, the primary metabolites of plants are of great importance from the perspective of survival and nutrition. Dossou et al. [111] studied the metabolite profile platform of five tissues (leaves, fresh seeds, white and purple flowers, fresh seed coat), based on ultrahigh performance liquid chromatography-mass spectrometry. A total of 776 metabolites belonging to different categories were identified qualitatively and quantitatively, and the composition of metabolites in the different tissues was significantly different. Among them, 238 key differential metabolites were screened for annotation and enrichment analysis, and it was found that flavonoid biosynthesis, amino acid biosynthesis and phenylpropane biosynthesis were the main different regulated pathways. In addition, Song et al. [50] detected primary metabolites in 412 sesame accessions using gas chromatography–mass spectrometry and identified 45 metabolites. Combined with genomic analysis and GWAS, 10 key candidate genes were found from the variation of fatty acid, asparagine, monoacylglycerols and sucrose contents. Brigante et al. [112] preliminarily identified 44 polyphenol compounds in different seeds through HPLC-DAD-ESI-qTOF (MS/MS), including 1 organic acid, 1 amino acid, 2 flavanones, 4 flavonols, 16 hydroxycinnamic acids and 20 lignans. In fact, lignans are the most important compounds for sesame, while flaxseeds show a variety of compounds with no major structural groups. Sesamin and sesamolin are the main lignans of sesame, and their percentages are 0.2~0.5% and 0.1~0.3%, respectively. In recent years, lignans have attracted the great attention of the world, especially in their superior antioxidant properties and remarkable health care effects, such as promoting ethanol metabolism or liver detoxification, regulating blood lipids and as an anti-cancer agent [113,114].

## 7. The Challenges of OMICS Approaches for Sesame Genetic Improvements

It is an opportunity and a challenge for the development of sesame genetics and breeding. The Central Rural Work Conference in 2021 pointed out that it is necessary to adjust the structure and expand the planting of oil crops, and implement the project to improve the production capacity of oil crops. At the end of 2020, the oil production in China reached a new historical high, and the planting area and total output of sesame among characteristic oil plants increased steadily. The traditional breeding of sesame includes system selection and hybridization breeding, heterosis breeding and mutation breeding [115]. However, system selection has a long cycle, poor predictability, low selection efficiency; mutation breeding also has several shortcomings, such as a low frequency of beneficial mutations, difficulty in controlling the direction and properties of the variation, and difficulty in inducing and identifying micro mutations. In addition, researchers have also tried to use cell engineering technology to modify some of the biological characteristics of cells to improve and create new varieties, but there is no variety of sesame bred by cell-engineering technology. Therefore, scientists proceed with molecular breeding technology, hoping to further improve the yield, quality and resistance of sesame varieties. Molecular breeding refers to the integration of modern biotechnology into classical genetic and breeding methods under the guidance of classical genetics, modern molecular biology and molecular genetics theory, and the combination of phenotype and genotype screening to cultivate new varieties. In addition, molecular breeding mainly includes molecular marker-assisted selection breeding and transgenic breeding. In recent years, a variety of molecular markers suitable for sesame research have been developed, such as SSR, RAPD, AFLP, SCAR, SRAP, SNP and InDel, but these markers are currently utilized for the genetic diversity analysis of sesame.

In general, the study of breeding technology and the breeding of new varieties in sesame have made a great progress, but there is still a certain gap compared with other crops, such as rice. The research on the molecular breeding of sesame is obviously backwards, and many problems need to be further studied and solved.

(1) Strengthen the development of markers related to the key agronomic traits of sesame: there are few markers related to important agronomic traits (such as resistance, yield and quality), which greatly limits the application of molecular marker-assisted selection in breeding. Therefore, we should excavate accurately and efficiently the markers that are closely linked to complex agronomic traits from several aspects: in-depth excavation of the genomic variation to obtain structure variation materials; improvement of the algorithm of GWAS or QTL to increase the detection force, such as a multi-sites GWAS method for detecting the rare sites. In addition, the existing molecular markers have a poor stability and low genetic effect in breeding. We need to improve the technology to really apply the effective molecular markers in sesame breeding.
(2) Analyzing the genetic mechanism of complex agronomic traits based on multi-omics: by combining the data of genomics, transcriptome, proteome and metabolomics, it the regulatory genes of complex agronomic traits could be revealed, their mechanism of actions, regulatory network and metabolic pathways could be clarified, and a theoretical basis and gene resources for modern molecular breeding (transgenic or gene editing) could be provided.
(3) Tightening modern biotechnology research and combining it with conventional breeding: the research of sesame cell engineering technology should be further strengthened to make it widely used in sesame breeding practice. The study of molecular marker-assisted selection breeding technology system should be further enhanced, and the obtained markers should be gradually applied to breeding practice. We should tighten the research on the transgenic technology of sesame disease resistance and stress-resistant genes, as well as the research on the heredity and safety evaluation of transgenic plant traits. In addition, we should strengthen the combination of cell engineering breeding, molecular breeding and conventional breeding, pay attention to the research of basic breeding theory and efficient breeding technology, and gradually move towards molecular design breeding.

## 8. Conclusions and Prospects

With the rapid development of sequencing technology, the number of relative studies on sesame genomics is also increasing. The completion of the sesame genome assembly indicates that significant progress has been made in sesame research, and the development of its omics studies has been presented. At present, the genomes of some sesame varieties have been sequenced, and high-density genetic linkage maps and pan-genomes have been constructed. This makes the genome resequencing and association analysis easier in sesame study, and provides support for the subsequent analysis (search for structural differences among genomes, the insertion and deletion sites, structural variation sites and copy number variation sites, etc.). In fact, the study of methylation can better link the genotype and epigenetic genotype. Especially under various abiotic stress conditions, it can help people to clearly understand the gene level changes in sesame varieties under different environmental conditions. Transcriptomics have been widely utilized in the study of abiotic stress, biotic stress, organ development, and noncoding RNAs. It focuses on exploring the differences in the gene expression level caused by slight changes in environmental conditions at the transcriptional level, and then how the function of the genes is affected. Although the proteome and metabolome have been less studied in sesame, researchers hope to use this information to obtain excellent genes that are related to oil synthesis and anti-oxidation, increase the breeding technology in sesame, improve the quality of sesame varieties, and understand the physiological mechanisms of some important traits in sesame.

Genomics research has promoted the fast development of methylome, transcriptome, proteome and metabolome, and has become the main method of revealing the potential mechanism of sesame genetic diversity, plant development, tissue and organ differentiation, and plant adaptation to biotic and abiotic stresses. Using this useful information, people hope to find the genes of drought resistance, salt tolerance and other important traits, the genes of sesame capsule cracking, or the genes of high oil and yield, and cultivate these varieties through molecular level experiments, which can be applied in practical production. In addition, we also hope that this information can be used to analyze the regulatory network of controlling oil or nutrients. In addition, the combination of multi-omics has enabled a deeper understanding of the mechanisms behind the complex structure of many agriculture-related phenotypic traits, enabling the large-scale identification of more genes and signals that are related to biological processes; the development of this research will lay an important foundation for the genetic breeding and molecular improvement technology of sesame.

## Figures and Tables

**Table 1:** The existing sequenced sesame genomes.

Cultivar	Zhongzhi 13	Zhongzhi 13	Swetha	Yuzhi 11	Baizhima	Mishouzhima	Baizhima	Xiaozihei
Type	Modern cultivar	Modern cultivar,	Modern cultivar	Modern cultivar	Landraces	Landraces	ND	ND
Genome size (Mb)	274	292	340	211	267	254	309	305
Technology	Illumina	PacBio/Hi-C	Illumina	Illumina	Illumina	Illumina	PacBio/Hi-C	PacBio/Hi-C
Contig N50 (kb)	52.2	1064.3	11.5	17.9	47.3	47.9	13,482	21,279
Scaffold N50 (Mb)	2.10	20.52	0.02	0.32	-	-	23.37	17.01
GC content (%)	35.22	-	35.00	35.10	-	-	35.44	35.93
Coding gene	27,148	28,406	41,859	26,022	31,558	30,995	24,345	25,265
Average length per gene (bp)	3171	-	4032	3623	3673	3700	3422	3112
Reference	[4]	[5]	[6]	[6]	[6]	[6]	[7]	[8]

ND: no data.

**Table 2:** The summary of chloroplast coding genes in sesame.

Gene Category	Genes
Photosystem I	*psaA, psaB, psaC, psaI, psaJ*
Photosystem II	*psbA, psbB, psbC, psbD, psbE, psbF, psbH, psbI, psbJ, psbK, psbL, psbM, psbN, psbT, psbZ*
Cytochrome	petA, * petB, * petD, petG, petL, petN
ATP synthase	atpA, atpB, atpE, *atpF, atpH, atpI
Rubisco	*rbcL*
NADH dehydrogenase	* ndhA,^§^ ^,^ * ndhB, ndhC, ndhD, ndhE, ndhF, ndhG, ndhH, ndhI, ndhJ, ndhK
Ribosomal protein (large subunit)	*^§^* ^,^ * rpl2, rpl14, * rpl16, rpl20, rpl22, ^§^ rpl23, rpl32, rpl33, rpl36
Ribosomal protein (small subunit)	*rps2, rps3, rps4, ^§^ rps7, rps8, rps11, ^§^* ^,^ *rps12, rps14, rps15, rps16, rps18, rps19
RNA polymerase	rpoA, rpoB, * rpoC1, rpoC2
ATP-dependent protease	* clpP
Cytochrome c biogenesis	*ccsA*
Membrane protein	*cemA*
Maturase	*matK*
Conserved reading frames	*ycf1, ^§^ ycf2, ycf3, ycf4, ^§^ ycf15*
Translational initiation factor	*infA*
Pseudogenes	*accD*

*^§^* Gene completely duplicated in the inverted repeat. * Gene with intron(s).

**Table 3:** The status of genetic linkage map in sesame.

Populations	Population Size	Number of Markers	Linkage Group Number	Total Length (cM)	QTL Manpping Reported	Reference
F2 population, COI1134 × RXBS	96 lines	8 EST-SSR, 25 AFLPs, 187 RSAMPLs	30	936.72	-	[24]
F2 population, COI1134 × RXBS	260 lines	30 EST-SSRs, 50 AFLPs, 573 RSAMPLs	14	1216.00	seed coat color	[25]
F6-RIL population, Zhongzhi 13 × Yiyangbai	206 lines	70 polymorphic SSRs, SRAPs and AFLPs	15	592.4	waterlogging tolerance	[26]
F8-RIL population, Zhongzhi 13 × ZZM2748	548 lines	424 SSRs	13	1869.80	charcoal rot resistance; sesamin and sesamolin content	[27,28]
F2 population, Zhongzhi 13 × Shandong Jiaxiang Sesame	107 lines	1233 SLAFs	15	1474.87	-	[29]
F8-RIL population, Miaoqianzhima × Zhongzhi 14	224 lines	1190 SNPs, 22 SSRs, 18 InDels	14	844.46	yield-related traits	[21]
F8-RIL poplation, Zhongzhi 13 × ZZM2748	430 lines	1522 bins	13	1090.99	plant height, seed coat color	[34]
F6-RIL population, 95-223 × 92-3091	91 lines	420 SNPs, 12 SSRs	13	914	-	[35]
BC1 population, Yuzhi 4 × BS	300 lines	9378 SLAFs	13	1974.23	basal branching habit, flowers per leaf axil	[36]
BC1 population, Yuzhi 4 × BS	150 lines	3528 SLAFs	13	1312.52	yield-related traits	[32]
F2 population, Gaoyou 8 × Ganzhi 6	122 lines	2159 SNPs	13	2128.51	seed-related traits	[30]
F2 population, Muganli-57 × PI 599446	120 individuals	782 SNPs	13	697.3	capsule shattering trait	[33]
F5-RIL population, Goenbaek × Osan	90 lines	1657 SNPs, 5 SSRs	13	883.37	phytophthora blight resistance	[31]
F2 population, Yuzhi DS899 × JS012	302 lines	3041 bins (30,193 SNPs)	13	2981.28	determinacy trait	[37]
F2 population, cl1 × USA (0)-26	130 lines	425,661 SNP/InDel variants	13	-	curly leaf and indehiscent capsule traits	[38]
F2 population, S-91 × S-297	149 lines	2339 bins (3030 SNPs, 16,279 InDels)	16	1497	yield components, seed mineral-nutrients	[39]
F2 population, Yuzhi DS899 × JS012	120 individuals	22,375 bins (380,544 SNP/InDel markers)	13	1576.14	seed coat color	[41]
F9-RIL population, Jinhuangma × Zhushanbai	180 lines	1354 bins (538,090 SNP/InDel variants)	13	1295.45	PEG-induced drought tolerance	[40]

**Table 4:** The applications of genome-wide association studies in sesame.

Traits	Number of Accessions	Number of QTN	Number of Candidate Genes	Reference
The 56 agronomic traits: oil content, fatty acid biosynthesis and yield	705	549	46	[44]
drought/salt tolerance	490	9/15	13/27	[45]
seed yield-related	705	547	48	[47]
drought tolerance	400	19	102	[46]
tocopherol content	96	1	1	[51]
seven root traits	327	19	32	[48]
morpho-agronomic traits	184	50	20	[52]
phytophthora blight resistance	87	29	34	[31]
seed coat color	366	224	92	[53]
melatonin content	450	3	14	[49]
primary metabolite content	412	433	10	[50]
phytosterol contents	402	33	37	[54]
fatty acid composition and oil content	400	43	20	[55]
specific lignans	410	89	10	[56]

QTN: quantitative trait nucleotides.

**Table 5:** The summary of omics studies on stress response in sesame.

Stress	Varieties	Tissues	Descriptions	Reference
Salt stress	WZM3063 (ST), ZZM4028 (SS)	shoot of seedling	Transcriptome and metabolome profiles in the seedlings of salt-tolerant and sensitive sesame genotypes were performed in the early phase of salt stress.	[76]
	WZM3063 (ST), ZZM4028 (SS)	shoot of seedling	miRNAs and their targets were identified from two contrasting sesame genotypes by a combined analysis of small RNAs and degradome sequencing.	[77]
Drought stress	ZZM0635 (DT), ZZM4782 (DS)	root	Decipher the response of tolerant and sensitive genotypes to progressive drought and rewatering based on transcriptome.	[78]
	ZZM3330 (DT), ZZM3743(DS)	leaf	Transcriptional and metabolic profiling in two sesame genotypes with contrasting ability to cope with drought stress.	[79]
	TEX-1 (DT), VEN-1 (DS)	root	Transcriptome analysis of two sesame genotypes with contrasting responses under PEG-induced osmotic stress.	[80]
Waterlogging stress	Zhongzhi 13 (WT), ZZM0563 (WS)	root	RNA-seq-based analysis between waterlogging-tolerant and -susceptible genotypes.	[81]
	ZZM2541 (WT), Ezhi3 (WS)	root	High-resolution temporal transcriptome analysis of two contrasting sesame genotypes over a 48 h period for waterlogging and drainage treatments.	[82]
Heat stress	Taizhi3 (HT), SP19 (HS)	leaf	Transcriptome analysis of two sesame cultivars with different heat tolerance.	[83]
Disease stress	Yuzhi 11 (DT), RXBS (DS)	seedlings	Transcriptome profiles of resistant and susceptible sesame germplasm resources inoculated with *Fusarium oxysporum* f. sp. *Sesami.*	[84]
	GT-10 (DT), RT-373 (DS)	root	Transcriptome analysis of resistant and susceptible sesame genotypes during *Macrophomina phaseolina* infection.	[85]

**Table 6:** Summary of transcriptome-based organ development studies in sesame.

Traits	Varieties	Tissues	Number of DEGs	Number of Candidate Genes	Reference
Oil content	ZZM4728, ZZM3495, ZZM2161	seeds, carpels	794, 1807, 528 and 1667 of DEGs at 10, 20, 25,30 DPA	23 sesame homologous lipid genes	[86]
Seed coat colors	Zhongfengzhi 1, Zhongzhi 33	seed	the maximum DEGs at 11 DPA (20,253)	20 genes	[87]
Seed coat colors	Yuzhi DS899, JS012	seed	2148, 5176, 3725, 2984 and 5115 of DEGs at 5, 10, 15, 20,25 DAF	28 genes	[41]
Oil content and fatty acid composition	Zhongzhi 16, Mishuozhima	seed	8404 DEGs	20 genes	[55]
Leaf size	Zhongzhi 13, ZZM2289	leaf	-	26 genes	[88]
Root size	Baizhima, 697	root	1831 and 1066 up and down regulated genes	10 genes	[89]
Male sterility	W1098A, W1098B	flower buds	1502 DEGs	49 homologous genes	[90]

## Data Availability

Not applicable.

## Abbreviations

DEG	Differentially expressed gene
IR	Inverted repeats
LSC	Large single copy
SSC	Small single copy
ISSR	Simple inter-sequence repeat
AFLR	Amplified fragment length polymorphism
SRAP	Sequence-related amplified polymorphism
SSRS	Simple sequence repeats
SNP	Single nucleotide polymorphism
INDEL	Insertion/deletion
LG	Linkage groups
SLAF-SEQ	Specific length amplified fragment sequencing
RAD-SEQ	Restriction-site associated DNA sequencing
GBS	Genotyping by sequencing
WGS	Whole-genome resequencing
WGBS	Whole genome bisulfite methylation sequencing
OXBS-SEQ	Oxidative bisulfite sequencing
RPBS	Reduced representation bisulfite sequencing
MEDIP-SEQ	Methylation DNA immunoprecipitation sequencing
HELP-SEQ	HpaⅡtiny fragement enrichment by ligation-mediated PCR
HPI	Hour post inoculation
DPA	Days post-anthesis
DAP	Differential abundant proteins
